# Resistance narratives in patients' accounts of a mandatory pre-operative health optimisation scheme: A qualitative study

**DOI:** 10.3389/frhs.2022.909773

**Published:** 2022-07-29

**Authors:** Isobel Avery-Phipps, Catherine Hynes, Christopher Burton

**Affiliations:** Academic Unit of Primary Medical Care, University of Sheffield, Sheffield, United Kingdom

**Keywords:** resistance narrative, preoperative health optimisation, smoking cessation, weight loss, preoperative period, general practice

## Abstract

**Background:**

Pre-operative Health Optimisation is the engagement of patients in health behavior change, such as smoking cessation and weight reduction prior to surgery. Programmes which routinely delay surgery while some patients undergo preoperative optimisation are increasingly used within the UK. Advocates of this approach argue that it reduces perioperative risk and encourages longer term change at a teachable moment. However, critics have argued that mandatory preoperative optimisation schemes may perpetuate or exacerbate inequalities.

**Aim:**

To understand patients' experience of a mandatory preoperative optimisation scheme at the time of referral for elective surgery.

**Design and setting:**

Qualitative interview study in one area of the UK.

**Method:**

Participants were recruited through GP practices and participating weight-loss schemes. Data was collected from nine semi-structured face-to-face interviews. Thematic analysis was informed by the concept of narratives of resistance.

**Results:**

Four forms of resistance were found in relation to the programme. Interviewees questioned the way their GPs presented the scheme, suggesting they were acting for the health system rather than their patients. While interviewees accepted personal responsibility for health behaviors, those resisting the scheme emphasized that the wider system carried responsibilities too. Interviewees found referral to the scheme stigmatizing and offset this by distancing themselves from more deviant health behaviors. Finally, interviewees emphasized the logical contradictions between different health promotion messages.

**Conclusion:**

Patients described negative experiences of mandatory pre-operative health optimisation. Framing them as resistance narratives helps understand how patients contest the imposition of optimisation and highlights the risk of unintended consequences.

## Introduction

Pre-operative health optimisation is a process of facilitating health behavior change to reduce surgical risk before a patient has an operation (1). It is most commonly used to support weight reduction and smoking cessation and has been widely promoted by commissioners of specialist services (2). Within the UK in 2018, 80% of Clinical Commissioning Groups (CCGs) employed some form of preoperative policy for weight management, and 35% employed a policy for smoking cessation (2).

The evidence for pre-operative health optimisation is of variable quality. Smoking cessation for a period of 8 weeks or more pre-operatively is associated with better perioperative outcomes (3) but the evidence for weight loss is weak, particularly unless the BMI is >35 (4, 5). While a few case series have reported sustained healthy behavior post operatively (6), there have been few robust studies of this. Economic drivers for preoperative optimisation have been described both from a commissioning perspective (paying for less operations) (2) or a hospital cost perspective (excluding patients most likely to have prolonged stays) (4).

Preoperative optimisation can be framed positively in terms of both reducing surgical risk and providing long term benefit through the adoption of change at a “teachable moment” (6), However, a more critical view is that it is used as an implicit form of rationing: smokers and overweight people may be seen as easy targets for NHS savings (2). As obesity and smoking show strong socioeconomic gradients, blanket enforcement of restriction for surgery based on these may widen health inequalities (7) and reduce patient autonomy (4). Little is known about patient perceptions of mandatory schemes: one recent small qualitative study (8) (7 patients) found largely positive views, however. However, the study did not report the socioeconomic status of participants and did not examine the ways that such schemes may deepen inequalities.

One approach to understanding how people resist health-promoting messages is through the concept of “resistance narratives” (9). These represent accounts which indicate agency through resistance while avoiding direct, or symbolic, confrontation with authority or elite norms (10). Such narratives are widely seen in the context of power and relations of social inequality, asymmetry and force (11). Resistance does not only reject subordination by those in power, but does so by challenging the ideologies that support that subordination (12).

We carried out a qualitative interview study to explore the views of people who had been referred by their GPs to a mandatory preoperative optimisation scheme, particularly focusing on areas of high socio-economic deprivation and using resistance narratives as an interpretive lens.

## Methods

### Setting

The study took place in an area of Yorkshire, England, served by one Clinical Commissioning Group (CCG) and one Acute Hospital Trust (AHT) which together were responsible for primary and secondary care services in the area. The CCG introduced a mandatory scheme in January 2018. The scheme applies to patients over 18 who require a referral to elective surgical services, and who have a BMI over 30 and/or are a current smoker. Instead of referring to the specialist service, GPs are expected to refer these patients for a 6-month optimisation period where the patient can access pre-operative weight loss and/or smoking cessation services commissioned by the CCG. If a patient successfully achieves a BMI of 30 (or loses 10% of their body weight for patients who initially had a BMI over 35) and/or stops smoking during the health optimisation period, they can be referred to surgery sooner.

The study was approved by London—Camberwell St Giles Research Ethics Committee (REC reference: 18/LO/1714) and all participants provided written informed consent. Interviews took place between January 2019 and August 2019. Ethical considerations focused on ensuring a protective environment for participants to express negative views about the healthcare system.

### Participants

We invited adults who had been referred to the preoperative optimisation scheme (either because of obesity or smoking) between January 2018 and March 2019. We excluded patients who were housebound or who did not use spoken English for their usual healthcare. Patients from all stages of the preoperative optimisation pathway, from recent referral through to postoperative recovery, were eligible to participate in order to sample a range of experiences in the short time-frame of the study. We invited participants through their GP practices and through weight-loss services which delivered the preoperative optimisation scheme.

### Recruitment

Six practices used a computer search of patients who had been referred to preoperative optimisation by the practice. From the list of names they were each asked to invite between 20 and 40 patients who met our inclusion and exclusion criteria using a postal invitation pack with prepaid reply. Recruitment from weight-loss centers involved visiting center at times when they were running sessions for preoperative optimisation referred patients and discussing with patients directly.

Interested potential participants were contacted by telephone to further explain the study and ask about the level of participation in preoperative optimisation activities (from full to none) prior to arranging the research interview.

### Data collection

Data was collected through semi-structured face-to-face interviews lasting between 30 and 60 min. Interviews took place at the participant's local GP practice or weight-loss service. Interviews followed a topic guide designed to explore the participants' perspectives and experiences of preoperative optimisation at all stages of the process: from before they were referred to the scheme, through to what they envisioned for the future. Interviews were digitally recorded and transcribed for analysis which was conducted using nVivo (QSR International Pty Ltd).

### Researcher reflexivity

The primary researcher (IA-P) was a medical student; the supervisors (CDB and CH) were both GPs with experience of research and practice in socio-economically deprived settings. Discussions about the project and the problem being studied took place over the 6 months prior to data collection and during data collection and analysis, when meetings were held weekly to ensure adequate supervision and contribute to the analysis. At all stages of the research, team members reflected on their personal views and experiences in relation to mandatory health optimisation.

### Analysis

The analysis was carried out as a reflexive thematic analysis (13). We used both inductive (generation of new themes from the data) and deductive (applying existing theories) approaches in the analysis. The theoretical position was one of interpretivism, acknowledging that the analysis was an interpretation of participants accounts, which in turn are interpretations of events (14).

In the later stages of analysis, we drew on ideas of resistance narratives as a lens to understand participants' accounts. Broadly, resistance narratives are one way by which individuals or groups put forward rational or moral arguments which counter the prevailing discourses. They may act to justify non-adherence with expected norms (10). Resistance narratives have been used in understanding a range of phenomena in health, including health promotion across socioeconomic groups (9), the relationship between physician and patient (15), and identity management in relation to stigma for overweight women (16). We sought to use the idea of resistance narratives to understand participants accounts in ways that went beyond simple concepts of barriers and facilitators (17).

## Results

### Participants

GP practices sent 147 invitations resulting in 7 participants. A further 2 participants were recruited through their weight loss service provider. Six participants were women and three were men (Table 1). Six were aged between 55 and 70, with one older than 70 and 2 younger than 55. Eight of the nine had postcodes with greater than average socioeconomic deprivation for England. All had been prescribed preoperative optimisation services but only five had used them: two patients stated they had not been offered referrals to services and two had decided not to use the referral.

**Table 1:** Participant characteristics.

**Participant's name** ^*^	**Age bracket**	**Gender**	**Operation**	**Decile of IMD** ^**^	**Stage of preoperative optimisation at time of interview** ^***^
Ann	40–55	Female	Vascular	2	In POHO period
Barbara	55–70	Female	Orthopedic	2	POHO completed, waiting for operation
Jessie	<40	Female	Orthopedic	1	In POHO period
Leslie	55–70	Male	Hernia	1	Post-operative
Martin	55–70	Male	Orthopedic	5	POHO completed, waiting for operation
Michelle	55–70	Female	Orthopedic	4	In POHO period
Pat	>70	Female	Orthopedic	3	In POHO period
Sandra	55–70	Female	Orthopedic	9	POHO completed, operation not required
Stanley	55–70	Male	Orthopedic	5	Referred to POHO

* *Participants' names have been changed to preserve confidentiality*

** *Based upon participant's postcode.*

*** *Stages in chronological order: in POHO period; POHO completed, waiting for operation/operation not required; post-operative POHO, pre-operative health optimization; IMD, Index of Multiple Deprivation; 1, most deprived decile; 10, least deprived decile*.

Within the preoperative optimisation scheme 4 were referred for weight loss alone, and five for both weight loss and smoking cessation. Five of the nine were currently in their 6-month preoperative optimisation period while four had completed the optimisation period; of these one had undergone surgery, two were still waiting for their procedure, and one had elected to not have surgery.

### Overview of themes

Interviewees were divided in their enthusiasm for the scheme. Two individuals had engaged well-with the scheme and felt it had been successful for them.

As I said, for me it's been a life change: a total life changing experience which wouldn't have happened if I hadn't have come on the scheme in the first place (Martin).

However, the remaining seven regarded it more negatively. Participants mentioned generic themes relating to accessibility of services, and of feeling that their individual circumstances were not respected or their needs met (17). However, this analysis focuses on four themes relating to how patients described their acceptance of, or resistance to, the preoperative optimisation scheme. These are repositioning the GP, re-framing responsibility, distancing from deviance and conflicting logics.

### Repositioning the GP

Participants' first exposure to preoperative optimisation was through the GP at the time of referral. Descriptions of being told about the preoperative optimisation scheme were recounted in ways which suggested a difference from the normal doctor-patient relationship.

[I] said “Look I've had three injections, things aren't working out, I want to be referred back up to the hospital,” and bluntly he said “Well, I can do but I know for a fact they'll knock you back because I can see that you're overweight” (Martin).

Several participants indicated that they thought this was unusual behavior by their GP, for instance by suggesting the GP was acting out of character.

I thought: “What's up with her [the GP]? She woke up on the wrong side of the bloody bed?” [laughs] “Sorry, so did you say no then?” “Yes, I did.” Her face never altered. They look as if they're really mad that they've been told to do this (Pat).

In several accounts GPs were portrayed as doing what they had been told by “the system”:

It's like sometimes I feel as if they're only saying it because it's, like, because they've been reminded by a computer or the government or whatever. Or “you've got so-and-so on your book, give ‘um a kick up the backside” (Ann).

Together these indicate a process of positioning the GP as different from usual and as an instrument of “the system”.

### Re-framing responsibility

All participants indicated that they recognized their own responsibility for their health to some extent. This was most noticeable in the two people who had engaged actively with preoperative optimisation, one of whom described it as a “lightbulb moment” and the other as a timely “kick up the backside.” The participant from the more affluent area described how

With [the preoperative optimisation programme], there are things that we can do to improve [our] quality of life. And that isn't for others to do. I think people have to take responsibility sometimes for their own health and wellbeing. Be that smoking, stop smoking, be that, you know, lose weight, be that take more exercise (Sandra).

This statement was in the context of her describing how she had joined a fitness programme outside of the preoperative optimisation scheme and was living off a diet of special weight loss products, strictly following this brand's regimen.

This view of individual responsibility was also expressed by participants who had not engaged with preoperative optimisation.

People cause some of [the] problems what need surgery. I think that's why they're wanting people to stop smoking. People stop smoking and start losing weight—well it will be better for everybody really won't it. It would be much better for the person who's actually done it, for surgeons who's having to concentrate more on people who really are sick and haven't like put it on [themselves] (Jessie).

While Jessie's account is superficially similar to Sandra's it is provided from a third person perspective, providing distance from Jessie's own story. She then indicated that personal responsibility did not remove the need for individuals to be treated with respect.

But still that's not a reason for [doctors] to treat people like [pauses] like they're beneath them (Jessie).

Similarly, participants described how they accepted a degree of personal responsibility but the ability to enact that was conditional on appropriate resources

And it's alright government and all the health professionals saying “Oh you need to do this you need to do that, you've got to try this, you've got to try that.” We know that, the public know that. I know that! But sometimes it's not that easy … you're not going to do it no matter how much your good intentions are. I can't, d'ya know what I mean, I can't do it (Ann).

Others described preoperative optimisation as an attempt by services to shift responsibility for gaps in provision onto individual patients and compliance with preoperative optimisation as nothing more than a game to be played in order to have necessary surgery.

…if that's how they're keeping waiting list down because government, you know, the government can say “They're only waiting 6 week or whatever for their operation”… I feel like I've been blackmailed, and I've given up smoking before and I've started again, and I will give up again. [But my arthritis is] not going to improve until they do something about it, ‘til I have this surgery…. Whether I lose weight or not. It's not going to improve (Stanley).

In summary, participants embedded common beliefs about personal responsibility for health lifestyles to lend legitimacy to their opinions and stories (9), however many then emphasized responsibilities of “the system” to work with them.

### Distancing from deviance

Health promotion discourses, such as those of preoperative optimisation, contribute toward establishing normalities of what it means to be “healthy”: in doing so these discourses create categories of deviants (9). Patients described feeling “offended,” “upset,” or “angry” when being told they fitted into the category of patients who required preoperative optimisation.

For instance Leslie was only a few Kg over the preoperative optimisation threshold, had managed to lose the weight and had since had his operation, yet he still recalled the initial experience with his GP with anger.

Well it were them that said you'll have to lose a bit of weight before we can operate on you. Well I don't know why they said that because there are some people who are a lot bigger than me that had hernia problems… I could have given them a rollicking saying “Why have you told me to lose weight like this and I'm not big, or whatever, and look at them over there” (Leslie).

Through much of the interview Leslie emphasized in different ways the differences between him and others, echoing common views of obese people as unlikeable and having less self-discipline than thin people (16). His account used language to distance himself from this group and reduce the stigma that he feels from this event.

For others who accepted the label of being overweight and/or a smoker, the experience of preoperative optimisation reaffirmed their view that smokers or overweight people are treated and viewed as second-class citizens.

[I feel] let down really, because certain people in their lives if they choose to do drugs or be alcoholics they get seen, but when somebody's overweight I just think it's a different matter…With equality and diversity, you're not allowed to but when you're overweight that's the one people that seems to get labeled a lot (Michelle).

The experience of referral to preoperative optimisation felt stigmatizing and discriminatory to patients. Participants, in giving their accounts and maintaining their identity (18), used different means of distancing themselves away from the tarnished identity which was provided by the scheme.

### Conflicting logics

The accounts of participants who did not engage, or did not succeed, in meeting their targets were characterized by highlighting contradictory logics. The most pervasive of these was simultaneously stopping smoking and losing weight. All 5 of the patients who were expected to stop smoking discussed how it was impossible for them to lose weight at the same time. For instance, Barbara, who had managed to lose enough weight to be referred for her operation yet had continued to smoke since her referral to preoperative optimisation described going to the smoking cessation service:

So, I went to see a stop smoking lady—and she tell me I would put weight back on! So, it's a catch 22 situation. So, my opinion is they shouldn't expect you to do both …. because it's hard, it really is. It's: which is the worse of two evils. To me the better one is to lose weight—unless you've got a chest complaint or something like that (Barbara).

Here, Barbara does not just highlight the contradicting logics, she provides a rational resolution of the contradiction which is hard to disagree with. Others called on personal experience and advice from trusted doctors as a ‘loophole' (16).

They've tried getting me [to see] the stop smoking lot and [pauses] part of me wants to give up. Another part of me don't. I mean years back me doctor he said to me “I'm not being funny” he says “but I'd rather you be smoking than drinking,” because when I was younger … I'd drink a bottle of [whisky] before I'd even left the house” (Ann).

Only one patient, resisted the argument about benefits of losing weight and stopping smoking, which she did on the grounds of her age

Come on, how much fitter am I gonna be at eighty, flower, that can hardly even walk without her legs aching. You know, just think “What they on about?” (Pat).

These narratives which resist the logic of following the preoperative optimisation guidelines shows the vast array of health care messages available to patients (9) and the ways that patients can use these to undermine preoperative optimisation.

### Resistance narratives

Taken together these themes provide examples of “resistance narratives” (9). The themes of repositioning the GP, reframing responsibility and distancing from deviance all represent indirect forms of resistance. Pointing out conflicting logics challenges the underlying ideologies behind the preoperative optimisation scheme.

## Discussion

### Summary of main findings

Patients referred to a pre-operative health optimisation programme described their experiences of the scheme in ways which could be understood as resistance narratives. Those who resisted the scheme positioned themselves as facing a “system” which had altered their GPs' behavior, deflected societal failures onto individuals, and grouped them with less desirable others, while at the same time requiring that they achieve something irrational and unattainable.

### Strengths and limitations

The study sample comprised a small number of participants recruited over 6 months in one area. This represented a low participation rate which was a key limitation for data collection and analysis. This was despite multiple rounds of recruitment and expanding the scope of recruitment from GP practices to weight-loss providers. Further efforts to recruit patients were not possible due to limitations in time frame of the study. We were unable to explore why other invitees chose not to take part but the deliberately neutral tone of the study information meant (in relation to positive or negative views) meant that the study did not advertise itself as targeting individuals with a particular opinion.

While only two of the nine participants regarded the preoperative optimisation scheme positively (and both were from more affluent areas) the remaining interviewees all regarded it negatively and used one or more of the resistance narrative themes described here. Despite the small sample size this was at the lower bound of a recent assessment of sample size to achieve code saturation (19) and the later interviews showed no new themes in relation to resistance narratives. We were unable to interview the GP practices about the scheme due to the study's limited timeframe.

Although the study was limited to one geographical area, mostly characterized by relatively high socioeconomic deprivation, framing the results within a wider theoretical model of resistance narratives—which have been seen in a number of different settings—increases its transferability. In particular none of the themes reported here related to specifically local contexts which would reduce transferability of the findings.

### Relationship to existing research

Other qualitative studies have examined preoperative optimisation, yet few have focused upon schemes requiring mandatory referral. One study interviewed patients who were required to lose weight prior to bariatric surgery (20). Engaging with preoperative optimisation was perceived as playing the “ideal patient role” which would allow them to be approved for surgery. The authors describe this as “paying for surgical approval through weight loss.” This contrasted with studies of voluntary preoperative optimisation, where participants engaged due to their belief that behavior change would benefit their health and reduce their risks of operative complications (21–24).

One recent small study (8) (7 patients) reported on a mandatory scheme similar to that studied here, but in more positive terms. However, the study did not report the socioeconomic status of participants and did not examine the ways that such schemes may deepen inequalities. Health promotion models, and “teachable moments” are two approaches taken to understanding preoperative health optimisation. The COM-B model (25), suggests that capability, opportunity, and motivation are all needed for behavior change in line with health policies. While these elements could be observed in our data in relation to engagement with POHO, they do not capture the resistance narrative elements of subversiveness to the scheme, and “the system” in which it had been created. The concept of teachable moments (26) featured in two qualitative studies on voluntary preoperative health optimisation schemes (21, 24). For the two participants—who engaged in health optimisation—it was applicable. However, other participants, rejected the “teaching” which they viewed as opportunistic.

There has been limited study of resistance narratives to health promotion messages. Merrild described resistance to health promotion messages among both affluent and poor Danes (9). In both, the resistance related to the power of the health promotion discourse, however the affluent participants used their resistance to sustain personal choices for living well, while poor participants described their resistance in terms of having to deal with illness and hardship. While the idea that professionals should exercise restraint in recommending health behavior change is not new (27, 28), our findings are a timely reminder of the hazards of blanket recommendation.

### Implications for practice, policy and research

The imposition of mandatory preoperative optimisation generates resistance narratives in some patients. These are likely to have consequences. First, accounts of resistance carry an emotional charge and demand a response (12): thus health professionals such as GPs are implicitly challenged by patients' resistance to show “who's side they are on.” Second, accounts of resistance may underpin non-engagement with the healthcare system by people with real needs; this has the potential to worsen health inequities. Third, as resistance narratives are shared, they can increase divisions across power divides such as between GPs and their patient communities. These resistances are not, therefore, simple inertia, or a neutral barrier to change. Rather they are active counter-narratives which have unintended consequences such as undermining the sense of the GP being on the side of the community. Exploring the perspectives of mandatory preoperative health optimisation schemes for GPs and secondary care providers would strengthen our understanding in this field.

Given the level of uncertainty about clinical benefits of preoperative optimisation, this study raises further doubts about mandatory schemes other than for smoking. In particular, the conflicting logics of simultaneously losing weight and stopping smoking necessitate a rethink about setting achievable objectives.

## Conclusion

Patients described several negative experiences of mandatory pre-operative health optimisation. Framing these as resistance narratives helps understand the ways by which patients reject and disengage from health optimisation and highlights the risk of unintended consequences.

## Data availability statement

The raw data supporting the conclusions of this article will be made available by the authors, without undue reservation.

## Ethics statement

The studies involving human participants were reviewed and approved by London—Camberwell St Giles Research Ethics Committee (REC reference: 18/LO/1714). The patients/participants provided their written informed consent to participate in this study.

## Author contributions

CB planned the original study design which was developed by CB, IA-P, and CH. IA-P carried out the interviews and conducted the initial analysis. CB and CH also contributed to the analysis. The manuscript was drafted by IA-P and subsequently revised by IA-P, CH, and CB. CB acts as guarantor for the research. All authors contributed to the article and approved the submitted version.

## Conflict of interest

The authors declare that the research was conducted in the absence of any commercial or financial relationships that could be construed as a potential conflict of interest.

## Publisher's note

All claims expressed in this article are solely those of the authors and do not necessarily represent those of their affiliated organizations, or those of the publisher, the editors and the reviewers. Any product that may be evaluated in this article, or claim that may be made by its manufacturer, is not guaranteed or endorsed by the publisher.
